# Occurrence and characterization of quinolone resistant *Escherichia coli* from Norwegian turkey meat and complete sequence of an IncX1 plasmid encoding *qnrS1*

**DOI:** 10.1371/journal.pone.0212936

**Published:** 2019-03-11

**Authors:** Jannice Schau Slettemeås, Marianne Sunde, Charlotte Rosenberg Ulstad, Madelaine Norström, Astrid Louise Wester, Anne Margrete Urdahl

**Affiliations:** 1 Section of Food Safety and Emerging Health Threats, Department of Animal Health and Food Safety, Norwegian Veterinary Institute, Oslo, Norway; 2 Division of Infectious Disease Control, Norwegian Institute of Public Health, Oslo, Norway; 3 Section of Epidemiology, Department of Analysis and Diagnostics, Norwegian Veterinary Institute, Oslo, Norway; 4 Water and Sanitation Unit, Department for Public Health, Environmental and Social Determinants of Human Health, World Health Organization, Genève, Switzerland; Nankai University, CHINA

## Abstract

Plasmid-mediated quinolone resistance (PMQR) is frequent among *Escherichia coli* from various food products and animals in several countries. The objective of this study was to characterize quinolone resistant *E*. *coli* (QREC) from Norwegian turkey meat regarding resistance profiles, genetic mechanisms for quinolone resistance, genetic relatedness, and to investigate whether PMQR genes were present. In total, 78 QREC were isolated by a selective method from 156 samples throughout 2013. Isolates were subjected to susceptibility testing, characterization of resistance mechanisms, serotyping, phylotyping and multi-locus variable-tandem repeat analysis (MLVA). All 78 isolates were resistant to ciprofloxacin, while 77 were also resistant to nalidixic acid. The nalidixic acid sensitive isolate had a resistance profile indicating the presence of a PMQR gene. Both PCR and whole genome sequencing confirmed the presence of a 47 304 kb IncX1 plasmid containing the *qnrS1* gene. The mechanism conferring quinolone resistance in the remaining isolates was mediated by mutations in the quinolone resistance-determining region of the chromosomal *gyrA* gene and for most of the isolates also in the *parC* gene. Molecular typing by MLVA showed a high degree of genetic diversity, although four clusters dominated. Two clusters contained strains belonging to phylogroup D/serogroup O176, the third contained isolates of phylogroup B1/serogroup O19, whereas the fourth contained isolates of phylogroup B1/non-typeable serogroup. Isolates within the latter cluster had MLVA profiles identical to QREC isolated from day-old imported turkey parent animals investigated in a preliminary study at the Norwegian Veterinary Institute. This finding suggests that QREC obtained from turkey may have been introduced via import of breeding animals to Norway. This is the first time the *qnrS1* gene is described from *E*. *coli* isolated from Norwegian turkey meat. Compared to available *qnrS1* carrying plasmids in Genbank, the current IncX1 plasmid showed high degree of similarity to other IncX1 plasmids containing *qnrS1* isolated from both *Shigella flexneri* and *E*. *coli* found in different geographical areas and sources. To conclude, this study showed that mutations in *gyrA* and *parC* are the main mechanism conferring quinolone resistance in *E*. *coli* isolated from Norwegian turkey meat, and that PMQR has not been widely dispersed throughout the *E*. *coli* population in Norwegian turkey.

## Introduction

Fluoroquinolones (FQ) are listed by the World Health Organization as critically important antimicrobials (CIA) for the treatment of human infections [1]. In the Norwegian livestock production, the usage of FQ is very low [2]. Furthermore, the quinolone resistance among bacteria from production animals has been rare. This is well documented in the Norwegian monitoring programme for antimicrobial resistance in feed, food and animals (NORM-VET, http://www.vetinst.no/overvaking/antibiotikaresistens-norm-vet) [3]. However, in 2009, the NORM-VET programme discovered a small peak compared to earlier findings showing 8% quinolone resistance in randomly tested intestinal *E*. *coli* isolated from healthy broilers [4]. Furthermore, in 2012, there was a single finding of plasmid-mediated quinolone resistance (S1 Table) in an *E*. *coli* from chicken meat produced in Norway [5]. These findings raised questions of whether poultry was associated with a higher level of quinolone resistant bacteria than previously assumed and if PMQR had dispersed among *E*. *coli* in the poultry reservoir in the same way as seen in extended-spectrum cephalosporin (ESC) resistant *E*. *coli* [6,7]. Based on this, a selective method for the detection of quinolone resistant *E*. *coli* (QREC) was introduced into the NORM-VET programme in 2013 and applied to Norwegian produced turkey meat samples. This resulted in the finding of QREC in 78 out of the 156 investigated turkey unique meat samples (50%) [8].

*E*. *coli* has three main mechanisms conferring quinolone resistance; two of these are chromosomal mutations, whereas the third is plasmid-mediated. Chromosomal mutations occur either in genes involved in quinolone target enzymes like DNA gyrase and topoisomerase, or in genes encoding regulatory proteins for the expression of porins or efflux pumps, resulting in a lower intracellular accumulation of quinolones [9–11]. Accumulation of chromosomal mutations in the quinolone resistance-determining region (QRDR) of the *gyrA* and/or *parC* genes typically results in high-level resistance, with ciprofloxacin and nalidixic acid MIC values of ≥1 mg/L and ≥256 mg/L, respectively. In contrast, strains harbouring PMQR genes tends to exhibit ciprofloxacin MIC values between 0.06–1 mg/L and nalidixic acid MIC values between 8–32 mg/L as shown by Veldman *et al* 2011 [12]. Co-existence of PMQR and QRDR mutations, may, however, lead to high-level FQ resistance [11,13,14], and it is also shown that strains harbouring PMQR genes tend to mutate more easily in the QRDR [15,16]. Plasmid-mediated genes known to encode quinolone resistance are: *qnrA*, *qnrB*, *qnrC*, *qnrD*, *qnrS*, *qnrVC*, *aac(6’)-lb-cr*, *qepA*, and *oqxAB* [17,18].

PMQR is of special concern, as these genes may be located on conjugative plasmids that are able to spread horizontally between bacteria. Moreover, the plasmids carrying PMQR genes may also harbour genes conferring resistance to extended-spectrum cephalosporin’s (ESC) and/or resistance to other classes of antimicrobial agents [15,19–28]. PMQR genes have been shown to exist on transferable plasmids also carrying *bla*_CMY-2_ and/or *bla*_CTX-M_ found in *E*. *coli* isolated from Brazilian chicken meat [29]. In a Turkish study, *qnrB* and *qnrS* were found on extended-spectrum beta-lactamase (ESBL) carrying plasmids in *E*. *coli* from chicken meat [30].

The aim of the present study was to characterize 78 QREC isolated from Norwegian produced turkey meat regarding antimicrobial resistance profiles, genetic mechanisms for FQ resistance and genetic relatedness of the strains. Furthermore, the sequence of a PMQR plasmid was determined, a comparative analysis to published plasmids was performed, and the plasmids’ ability to conjugate was studied.

## Material and methods

### Bacterial isolates

A total of 78 QREC isolates originally isolated by selective cultivation from 156 turkey meat samples in the auspices of NORM-VET [31] were included in the present study. Meat samples were collected at retail throughout 2013 in the counties of Oslo, Akershus and Vestfold following a proportionate stratified sampling scheme, representing the market share of the small number of turkey meat production companies in Norway. The metadata regarding the samples are presented in S3 Table.

In short, five grams of turkey meat were cultured at 41°C in MacConkey broth for 24 hours before plating out onto two MacConkey agar plates (BD Difco™, Beckton, Dickinson and Company, Franklin Lakes, New Jersey, USA) containing 0.25 mg/L and 0.5 mg/L ciprofloxacin (Fluka™, Sigma–Aldrich, St. Louis, Missouri, USA), respectively. Typical *E*. *coli* colonies were subcultured on blood agar (Heart infusion agar, Difco Laboratories, Fischer Scientific, New Hampshire, USA) containing 5% bovine blood and incubated at 37°C for 24 hours, before being confirmed as *E*. *coli* by using a PCR to detect the *E*. *coli* specific *uidA* gene [32]. One isolate per unique meat sample were selected for further investigation and included in the present study.

### Susceptibility testing

Susceptibility testing was performed using a broth microdilution method on the Sensititre™ TREK EUVSEC plate (Trek Diagnostic System Ltd., United Kingdom). Isolates resistant to the cephalosporins; cefotaxime and/or ceftazidime were tested on the Sensititre™TREK EUVSEC2 plate in order to determine if the cephalosporin resistance was caused by ESBL or AmpC beta-lactamases. Epidemiological cut-off values (ECOFF) as recommended by the European Committee on Antimicrobial Susceptibility Testing (EUCAST, www.eucast.org) were used to categorize the isolates as susceptible or resistant. The fully susceptible *E*. *coli* ATCC 25922, and the cephalosporin-resistant strains *E*. *coli* K8-1 (*bla*_CTX-M_ positive) and *E*. *coli* K5-20 (*bla*_CMY-2_ positive), were included as quality controls.

### Characterization of resistance mechanisms

The isolates were subjected to PCR and sequencing of the *gyrA* gene by using a method previously described to detect mutations [33]. A 435 bp fragment of the *parC* gene was amplified using in-house designed primers; PARC.F 5'-ATATGGCAGAGCGCCTTGCGC-3’ and PARC.R 5'-CGAAGTTTGGCACCCAGTC-3 and annealing temperature was set to 53°C. PCR products were sequenced on a 3130*xl* or 3500 Genetic Analyzer (Applied Biosystems, Foster By, California, USA) using BigDye V3.1 cycle sequencing kit (Applied Biosystems). Sequences were processed and analysed in CLC Main Workbench (CLC Bio, QIAGEN, Aarhus, Denmark).

One isolate with a ciprofloxacin MIC above ECOFF and nalidixic acid MIC below ECOFF indicating the presence of PMQR genes [12], were subjected to PCR for the detection of; *qnrA*, *qnrB*, *qnrD*, *qnrS* and *aac(6’)-lb-cr* according to previously described methods [12,34,35]. The following positive control strains were included in the respective run: *Enterobacter cloacea* 03–577 (qnrA1+/CTX-M-9+), *Klebsiella pneumoniae* Kp15 (*qnrB1*+), *Salmonella enterica* Newport 2007601863–1 (*qnrB5*+), *E*. *coli* transformant p2007057 TF1 (*qnrD*+), *Salmonella enterica* Saintpaul 200860-85-2 (*qnrS1*+), and *Salmonella enterica* Typhimurium GSS-HN-2007-003 (*aac(6’)lb-cr*). *E*. *coli* ATCC 25922 was included as a negative control strain.

Isolates resistant to ESC and displaying a phenotype resembling an AmpC profile were subjected to real-time PCR in order to detect the plasmid-mediated AmpC gene *bla*CMY-2 and closely related variants [36]. Positive and negative control strains were included in the run. In case of negative results by *bla*CMY-2 real-time PCR, the isolates were further investigated for up-regulated AmpC production due to mutations in the promoter/attenuator region by a previously described method [37–39].

### Whole genome sequencing of PMQR isolate

In depth characterization of the isolate found to be positive for the PMQR gene *qnrS1* (NVI-2422) was performed by paired-end sequencing at the Norwegian Sequencing Centre on an Illumina HiSeq 2500 (Illumina Inc., San Diego, California, USA) using rapid run mode with a read length of 2x250bp. The whole genome sequencing (WGS) data were quality controlled (QC) by adapter and quality trimming using Trimmomatic [40], and assembled using SPAdes v3.11.0 using the “—careful” parameter [41]. Genetic characterization of the isolate was performed using the following online tools with default settings from the Center for Genomic Epidemiology (http://www.genomicepidemiology.org): ResFinder 3.0, PlasmidFinder 3.1, MLST 1.8 and SeroTypeFinder 1.1. The MLST scheme *Escherichia coli* #1 developed by Wirth et al 2006 was used to assign allele numbers [42].

A draft sequence of the plasmid was identified in an assembled scaffold containing the *qnrS1* gene sequence. The scaffold was 46710 bp in size and identified using CLC Main Workbench (CLC Bio, QIAGEN). To investigate if the scaffold could be circularized, PCR was performed followed by Sanger sequencing with primers hybridizing to both ends of the scaffold. Boiled DNA was used as template for the amplification of the region mentioned above. Primers were designed using CLC Main Workbench and the nucleotide sequence of the PCR product obtained was determined using BigDye Terminator v3.1/1.1 cycle sequencing kit and a 3500 Genetic Analyzer (both Applied Biosystems). The nucleotide sequence of the complete plasmid (pNVI2422, 47304 bp) was annotated by automated annotation using the Rapid Annotation using Subsystems Technology (RAST) server V2.0 [43] and PROKaryotic Annotation (Prokka) [44] together with manual annotation in CLC Main Workbench (CLC Bio, QIAGEN). The BLAST Ring Image Generator (BRIG) was used to visualize the comparison to other plasmids [45].

### Conjugation experiments

Conjugation experiments in liquid media were performed as described previously by Mo *et al*. [7] with *E*. *coli* OneShot™ cells with pCR™ II vector containing a kanamycin resistance gene (Invitrogen™, LifeTechnologies, Thermo Fisher Scientific Inc., Waltham, MA, USA) as the recipient strain. The experiments on solid media were performed using the same donor and recipient strains. Overnight cultures of recipient and donor was mixed 1:10 on Trypton Soy Agar (Oxoid, Thermo Fisher Scientific Inc.) and incubated at 37°C. Sampling of mating pairs in both experiments was performed after 4 or 24 hours by plating material from the mating pairs onto Mueller Hinton agar (Difco Laboratories, Fischer Scientific) containing 0.06 mg/L ciprofloxacin (Fluka, Sigma-Aldrich) and 50 mg/L kanamycin (Fluka, Sigma-Aldrich). The plates were incubated at 37°C for 24–48 hours. The morphology of the colonies was visually inspected for the typical characteristics of the recipient strain [46]. To confirm conjugative transfer, the transconjugants were subjected to PCR as previously described in order to verify phylogroup D of the recipient strain as well as the presence of the *qnrS* gene.

### Phenotypic serotyping

All isolates were O-serogrouped at the Norwegian Institute of Public Health using polyvalent and monovalent *E*. *coli* antisera according to the producer’s manual (https://www.sifin.de/download/T/ts2216/TS2216-GA-EN_2016_03_24_10_10.pdf). The isolates were tested against polyvalent antisera SSI pool 2 and pool 3 (SSI Diagnostica, Copenhagen, Denmark) and Anti-Coli I, Anti-Coli II, and Anti-Coli III (all from SIFIN Diagnostics gmbh, Berlin, Germany) (S5 Table). In addition, the following monovalent O antisera were used; SSI O19, O101, O176 and O8, and SSI OK O121 and O45 (SSI Diagnostica). Some of the specific monovalent antisera were included because of serogroup findings in ciprofloxacin resistant *E*. *coli* isolated from turkeys in the UK [47]. For testing against O antisera from SSI Diagnostica, a boiled culture was mixed with antiserum in a tube, incubated overnight in humid atmosphere at 52°C, and controlled for granulation (positive test). For testing against the polyvalent antisera from SIFIN (SIFIN Diagnostics gmbh) and monovalent SSI OK antisera (SSI Diagnostica), 20 μL antiserum was applied on a slide, together with 5–10 colonies from an overnight culture, mixed to a homogenous suspension and controlled for agglutination. If positive for agglutination in the polyvalent SIFIN antisera, the isolates were further tested against SIFIN monospecific antisera by microtiter plate agglutination and confirmed by agglutination in a Widal tube test, according to the producer’s manual (https://www.sifin.de/download/T/ts2216/TS2216-GA-EN_2016_03_24_10_10.pdf). NaCl was used as negative control for spontaneous agglutination.

### Determination of phylogroups

Phylotyping of all isolates was conducted using a multiplex PCR amplifying the three phylogenetic markers *chuA*, *yjaA* and TSPE4.C2, with primers as previously described [48]. Primers for the *gadA* gene were implemented as an internal amplification control. Isolates were grouped into the four phylogenetic groups A, B1, B2 and D according to the dichotomous decision tree for phylogenetic grouping established by Clermont et al 2000 [49]. An isolate belonging to the B2 group (*E*. *coli* 2003500827) exhibiting all four target genes was included as a positive control [46].

### Multi-locus variable-tandem repeat analysis

Multi-locus variable-tandem repeat analysis (MLVA) was conducted on all isolates using a previously described protocol by Lobersli et al. 2012 [50,51]. Altogether, ten loci were amplified (CVN001, CVN002, CVN003, CVN004, CVN007, CVN014, CVN015, CVN016, CVN017 and CCR001), by three multiplex and one singleplex PCR. The amplifications were mixed and multi fluorolabelled products were separated by capillary electrophoresis on a 3130*xl* Genetic Analyzer (Applied Biosystems). Each peak was normalized and identified according to colour and size using the Peak Scanner Software V2 (Applied Biosystems). An allele number was assigned based on fragment size. In case of no amplicons, allele number was designated as -2. Allele numbers were entered in BioNumerics as character values (BioNumerics version 6.6, Applied Maths NV, Sint-Martens-Latem, Belgium). The strain EDL933 was run as a positive control for the analysis.

Similarity between MLVA profiles were calculated using categorical coefficients and the unweighted pair group method with arithmetic mean (UPGMA) algorithm [52], and by the minimum spanning tree (MST) algorithm [53].

## Results

### Resistance to antimicrobial agents

Results of the susceptibility testing of the 78 QREC isolates are presented in Table 1. All isolates displayed MIC values above the ECOFF for ciprofloxacin at 0.06 mg/L. Except for one isolate, they also expressed resistance to nalidixic acid with MIC values above the ECOFF at 16 mg/L.

**Table 1:** Minimum Inhibitory Concentrations (MICs) and antimicrobial resistance in *Escherichia coli* resistant to fluoroquinolones (n = 78) isolated from turkey meat in Norway in 2013.

Substance	Resistance (%)	Distribution of MIC values (mg/L)															
		0.015	0.03	0.06	0.12	0.25	0.5	1	2	4	8	16	32	64	128	256	≥ 512
Ampicillin	57.7							1.3	23.1	16.7	1.3			57.7			
Azithromycin	NA								52.6	39.7	6.4	1.3					
Ciprofloxacin	100					38.5	3.8	1.3		9	41	6.4					
Nalidixic acid	98.7										1.3			3.9	19.2	75.6	
Gentamicin	15.4						39.7	41	3.9				7.7	7.7			
Tetracycline	34.6								65.4					11.5	23.1		
Colistin	0							91	9								
Sulfamethoxazole	60.3										38.5	1.3					60.3
Trimethoprim	34.6					60.3	5.1							34.6			
Chloramphenicol	10.3										89.7				1.3	9	
Cefotaxime	20.5					79.5		12.8	7.7								
Ceftazidime	20.5						79.5		9	11.5							
Meropenem	0		100														
Tigecycline*	0					97.4	2.6										

Bold vertical lines denote epidemiological cut-off values for resistance. NA, cut-off not defined by EUCAST. White fields denote range of dilutions tested for each antimicrobial agent. MIC values higher than the highest concentration tested are given as the lowest MIC value above the range. MIC values equal to or lower than the lowest concentration tested are given as the lowest concentration tested.

*Tentative ECOFF from the EURL-AR (www.eurl-ar.eu)

The most common co-resistance, found in this study, was resistance to sulfamethoxazole (60.3%), ampicillin (57.7%), tetracycline (34.6%), and trimethoprim (34.6%). Resistance to gentamicin and chloramphenicol were detected in 15.4% and 10.3% of the isolates, respectively.

Resistance to ESC occurred in 16 (20.5%) of the isolates. All isolates displayed a phenotypic profile corresponding to be an AmpC producer according to the EFSA summary report of 2018 [54] with MIC values to cefoxitin >8 mg/L, and cefotaxime and ceftazidime >1 mg/L. Further, all isolates were susceptible to meropenem with MIC values ≤0.03 mg/L and synergy with clavulanic acid was not detected.

### Genetic characterization

In 44 (56.4%) of the 78 isolates, quinolone resistance was caused by two mutations in *gyrA* at codon 83 and 87, and one mutation in *parC* at codon 80. In *gyrA*, the mutation Serine → Leucine (S83L), together with Aspartic acid → Asparagine at codon 87 (D87N) were detected. In *parC*, the mutation Serine → Isoleucine (S80I) was detected in 35 isolates, and Serine → Asparagine (S80R) in the remaining 9 of the 44 isolates. All the 44 isolates exhibited MIC of ciprofloxacin of ≥4 mg/L and nalidixic acid MIC of >128 mg/L. Single *gyrA* S83L mutation was detected in another 33 (42.3%) of the 78 isolates. Of the 33 isolates, one had additional S80R mutation in the *parC* gene and exhibited MIC of ciprofloxacin of 1 mg/L and nalidixic acid MIC of >128 mg/L. The remaining 32 of the 33 isolates exhibited MIC of ciprofloxacin of 0.25–0.5 mg/L and nalidixic acid of ≥64 mg/L and contained no mutations in the *parC* gene. One isolate did not contain any mutations in the *gyrA* nor *parC* genes and was suspected to carry a PMQR gene because of the MIC profile of ciprofloxacin of 0.5 mg/L and nalidixic acid of 8 mg/L.

Resistance to ESC was in 15 of the strains caused by nucleotide substitutions at position -42, -18 and +81 in the promoter/attenuator region of the chromosomally located *ampC* gene corresponding to promoter sequence variant 1 [38]. In the last strain, ESC was caused by nucleotide substitutions at position -18 and +81 corresponding to promoter sequence variant 11. All 16 ESC resistant *E*. *coli* isolates were also resistant to sulfamethoxazole and tetracycline, whereas 12 were simultaneously resistant to gentamicin.

Compilation of results from serotyping and phylotyping showed that approximately 45% of the isolates grouped into serogroup O176/phylogroup D (n = 14, 17.9%), serogroup O19/phylogroup B1 (n = 13, 16.7%), serogroup O8/phylogroup B1 (n = 3, 3.8%), and serogroup O8/phylogroup A (n = 1, 1.3%), Table 2 and S3 Table. The remaining 47 (60.3%) isolates could not be allocated to any of the O-groups included in the test panel using phenotypic serotyping, although one of them was grouped into O2:H5 using WGS data and belonged to phylogroup B2. The 46 (59.0%) non-typeable isolates were grouped in the following phylogenetic groups: D (n = 34, 43.6%), B1 (n = 10, 12.8%), B2 (n = 1, 1.3%) and A (n = 1, 1.3%).

**Table 2:** Characteristics of the four dominating clusters regarding phylogroup, phenotypic serogrouping, resistance profile and mutations in *gyrA* and *parC*.

Cluster	ID	Number of isolates	Phylo-group	Phenotypic serogrouping	Resistance profile^a^	Mutations in *gyrA*^b^	Mutations in *parC*^c^
C1	3233	1	D (n = 29)	nt	CIP, NAL, SUL, TMP, AMP, TET	S83L, D87N	S80I
	1218, 1318, 1386, 1520, 3055, 3078, 3606, 3788, 3866, 3867, 4931, 5151, 5157, 5683, 5684, 6107, 6126, 948	18		O176 / nt	CIP, NAL, SUL, TMP, AMP		
	5268, 6518	2		O176 / nt	CIP, NAL, TMP, AMP		
	3864	1		O176	CIP, NAL, TET,		
	2891, 2932, 3448, 4051, 5152, 5156, 649	7		O176 / nt	CIP, NAL		
C2	3057, 3058	2	D (n = 17)	O176 / nt	CIP, NAL, SUL, TMP, AMP, TET, CHL	S83L	None
	2147, 2502	2		nt	CIP, NAL, SUL, TMP, AMP	S83L, D87N	S80R
	5392	1		nt	CIP, NAL, TET		
	1664, 1665, 1808, 2311, 2574, 2575	6		nt	CIP, NAL		
	3604, 3687, 3743, 622, 623, 947	6		nt	CIP, NAL, SUL, TET, CHL		S80I
C3	2700, 2890, 3167, 3231, 3603, 3605, 3789, 3865, 4050, 5154, 6674	11	B1 (n = 15)	O19	CIP, NAL, SUL, AMP, TET, FOT, TAZ, FOX, GEN	S83L	None
	1410, 2701, 3054, 5320	4			CIP, NAL, SUL, AMP, TET, FOT, TAZ, FOX		
C4	2254, 2310	2	B1 (n = 7)	nt	CIP, NAL, AMP	S83L	None
	2424, 3937, 5348, 5600, 6376	5			CIP, NAL		

nt–non-typeable

^a^CIP, ciprofloxacin; NAL, nalidixic acid; SUL, sulfamethoxazole; TMP, trimethoprim; AMP, ampicillin; TET, tetracyclines; CHL, chloramphenicol; FOT, cefotaxime; TAZ, ceftazidime; FOX, cefoxitin

^b^S83L, Serine to Leucine at codon 83; D87N, Aspartic acid to Asparagine at codon 87

^c^S80I, Serine to Isoleucine at codon 80; S80R, Serine to Arginine at codon 80

### Plasmid characterization

**Fig 1:**
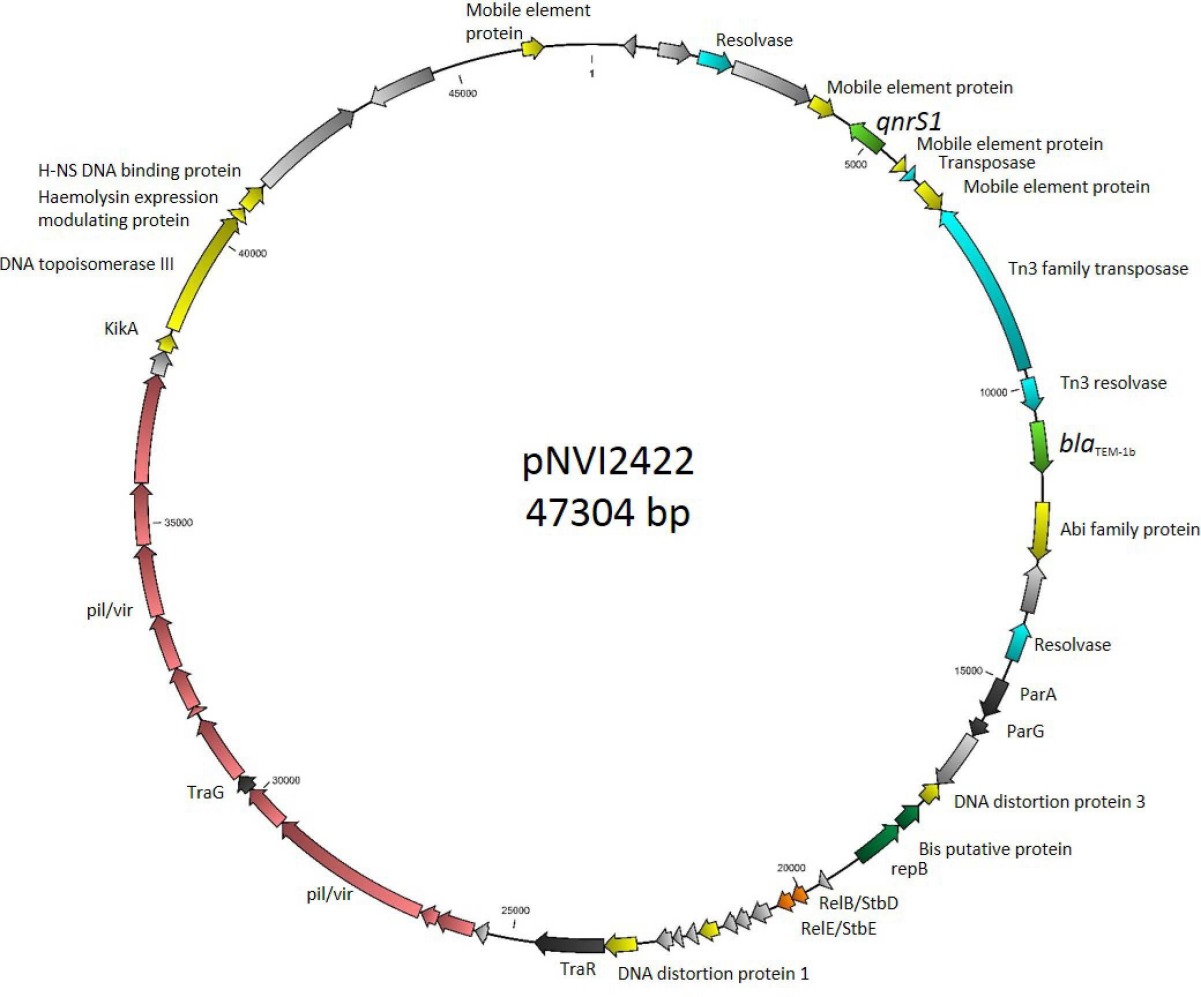
Illustration of the *qnrS1* bearing IncX1 plasmid pNVI2422. Dark grey colour indicates transfer associated genes, orange colour indicates plasmid stability system, green colour indicates antimicrobial resistance genes, red colour indicates pilus associated genes, dark green colour indicates replication associated genes, yellow colour indicates other proteins, light grey colour indicates hypothetical proteins, and light blue colour indicates transposon associated genes.

The isolate without mutations in the *gyrA* and *parC* genes was suspected to carry a PMQR gene and was further investigated. PCR detected the *qnrS* gene and conjugation experiments indicated the presence of a self-transferrable PMQR carrying plasmid. The genome of this isolate was sequenced, and the sequence data showed the presence of a *qnrS1* gene on a conjugative IncX1 plasmid (pNVI2422, Fig 1). The nucleotide sequence of pNVI2422 was reconstructed and closed by Sanger sequencing to close the gap in the original scaffold (46170 bp). The amplification and sequencing of the gap showed a ~1.3 kb fragment that was found on another small scaffold (1286 bp) of the WGS data.

**Fig 2:**
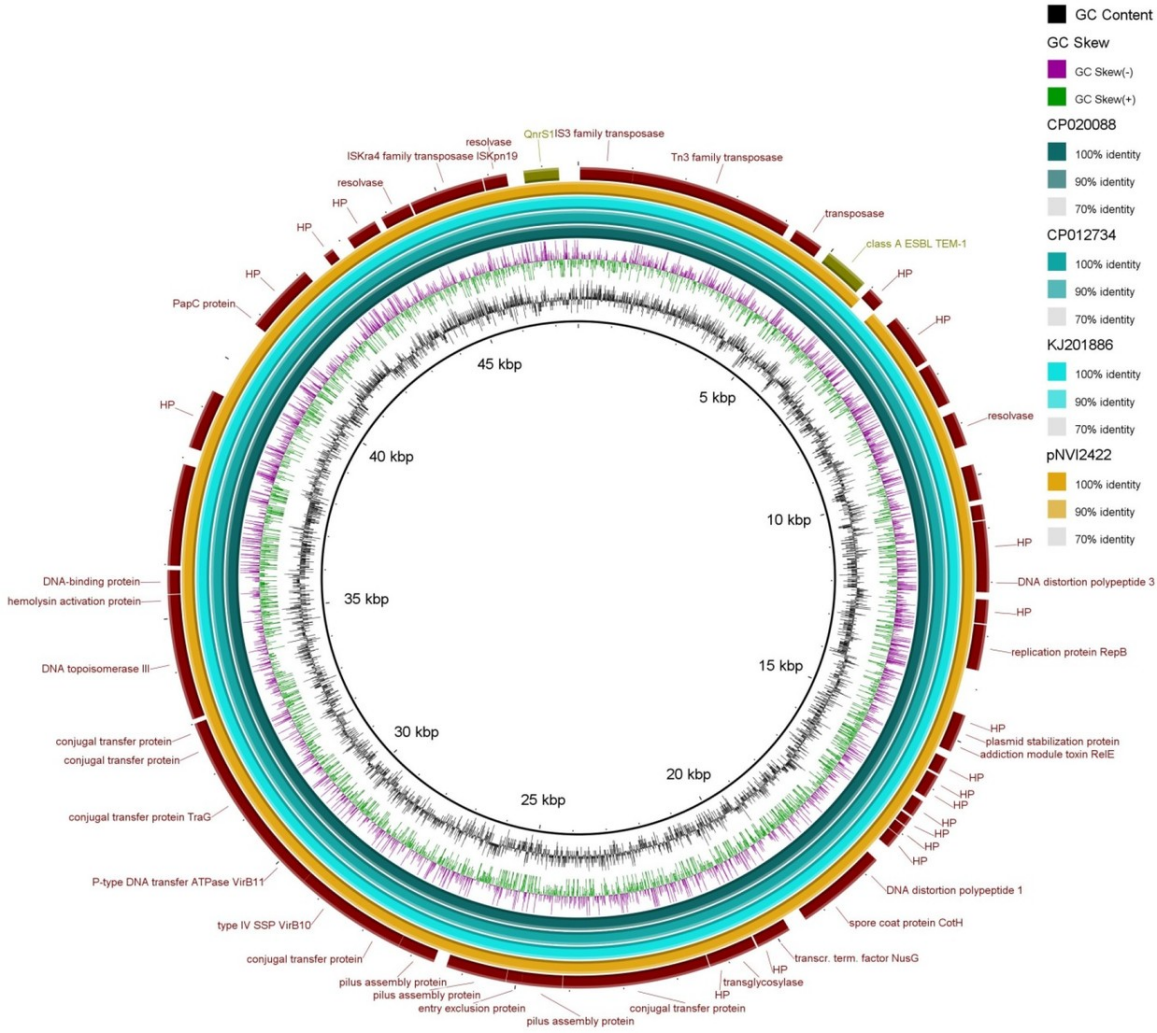
BRIG illustration of pNVI2422 compared to plasmids from three *Shigella flexneri* strains: 1a (CP020088), 4c (KJ201886), and 1a (CP012734). CP020088 was used as the reference genome.

**Fig 3:**
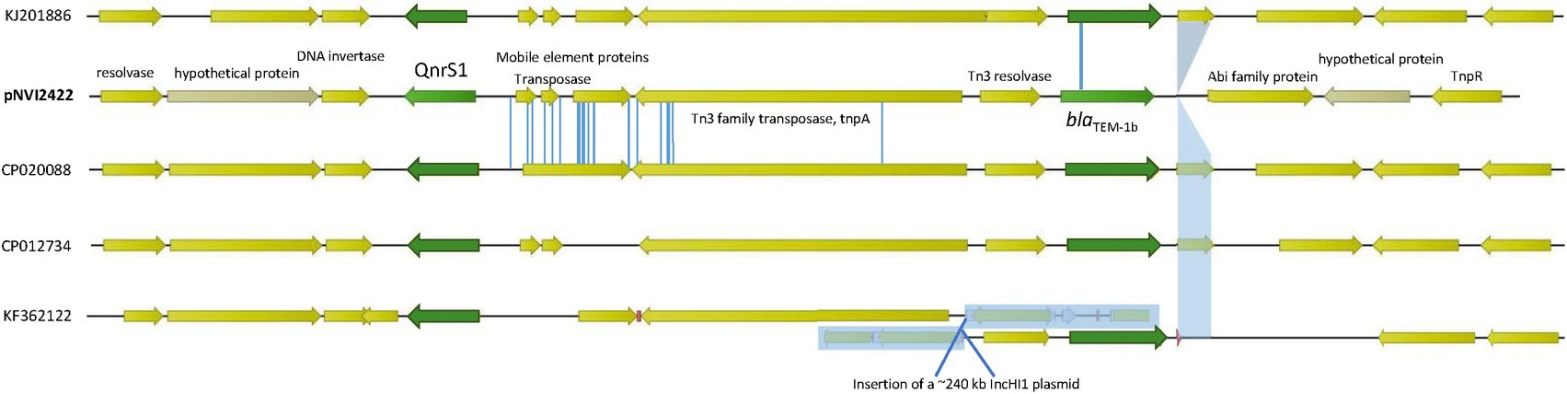
Illustration of the genetic load region of plasmid pNVI2422 compared to plasmids with high similarity. The annotation is performed on pNVI2422 and the resistance genes are in green colour. Blue areas and lines denote nucleotides missing or different compared to pNVI2422.

The plasmid had a total length of 47304 bp and carried *qnrS1* associated with an IS*2*-like element and a *bla*_TEM-1B_ gene on a Tn*3*-like transposon conferring ampicillin resistance. The strain carrying the plasmid belonged to sequence type (ST) 355, serogroup O2:H5 and grouped into phylogroup B2. Plasmid pNVI2422 was highly similar to plasmids previously described as shown in Fig 2. The three most similar plasmids were isolated from *Shigella flexneri* strains. Of these, three were isolated from human sources in China; accession number CP020088, KJ201886, CP012734. The plasmid pNVI2422 was also similar to a plasmid isolated from an *E*. *coli* strain from an equine source in the Czech Republic (acc. no. KF362122) [55]. The pNVI2422 IncX1 backbone consisted mainly of genes associated with conjugal transfer and transcription. In addition, the plasmid stability system *relBE/stbDE* was present on the plasmid backbone. The genetic load region illustrated in Fig 3 showed higher degree of similarity to the genetic load region on plasmids KF362122 and CP012734 as compared to KJ201886 and CP020088. The nucleotide sequence of pNVI2422 from strain 2422 was submitted to GenBank with accession number MH507589.

### Genetic relationship

**Fig 4:**
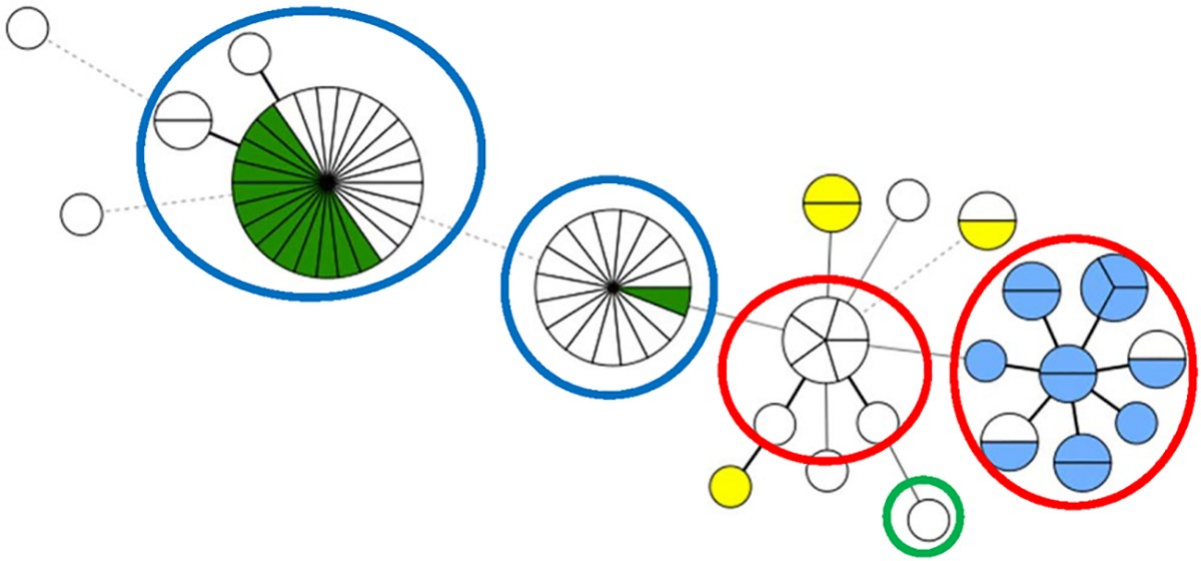
Minimum Spanning Tree of MLVA data showing the distribution of phylogroups and serogroups among the 78 QREC from turkey meat. The parts of the circles represent number of isolates. Dark green areas indicate serogroup O176, yellow serogroup O8, and blue serogroup O19. White areas indicate non-typeable isolates. Blue circles indicate phylogroup D and red circles phylogroup B1. Green circle indicates plasmid pNVI2422 (phylogroup B2, serogroup 02:H5).

A total of 23 MLVA profiles were detected among the 78 QREC isolates (S3 Table). Cluster analyses demonstrated four dominating clusters as shown in Fig 4. They contained isolates belonging to serogroup O176 or unknown O-group/phylogroup D isolates (n = 29, 37.2%), serogroup O176 or unknown O-group /phylogroup D isolates (n = 17, 21.8%), serogroup O19 or unknown O-group /phylogroup B1 isolates (n = 15, 19.2%), and one small cluster (n = 7, 9%) with phylogroup B1 isolates belonging to unknown O-group(s), respectively (Fig 4, Table 2). The isolates in the cluster comprising serogroup O19/phylogroup B1 were also resistant to ESCs. The PMQR isolate pNVI2422 belonged to phylogroup B2 and was genetically distinct from the other isolates.

## Discussion

This study indicates that mutations in *gyrA* and *parC* are the dominating mechanism for quinolone resistance in *E*. *coli* from the Norwegian turkey production. Although we only looked at isolates from 2013, the results indicate that PMQR has been less widely dispersed throughout the Norwegian turkey *E*. *coli* population compared to other animal populations [56].

We detected only one isolate in the present study that carried plasmid encoding quinolone resistance (pNVI2422), identified as *qnrS1* on a typical IncX1 plasmid backbone, in the same way as described by Norman et al [57]. IncX1 plasmids are widely distributed and have been detected in species of *Salmonella*, *Klebsiella*, *Shigella* and *E*. *coli* [55,58]. Dobiasova and Dolesjka [58] investigated a collection of *Enterobacteriaceae* from several sources (wildlife, municipal wastewater, humans, food producing animals, and captive animals from different continents) and a high occurrence of IncX1 plasmids containing *qnrS1* was found in *E*. *coli* strains with reduced susceptibility to ciprofloxacin (0.05 mg/L). These findings suggest that IncX1 plasmids containing *qnrS1* are successful and widely disseminated.

A study by Veldman et al published in 2011 [12] where they characterized PMQR suspected *Salmonella* and *E*. *coli* isolates from various food products and animal species, including turkey in thirteen European countries, showed that *qnrS1* was frequently identified. Like our result, one of the *qnrS1* positive *E*. *coli* isolate from broiler in the Dutch study was resistant to ESC [12,59]. In contrast to our results, however, the isolate from the Netherlands contained three different plasmids, all with a *qnrS1* gene together with an ESC resistance gene: *qnrS1* + *bla*_SHV-12_ on a 45 kb non-typeable plasmid, *qnrS1* + *bla*_CMY-2_ on an 80 kb IncK plasmid, and *qnrS1* + *bla*_CTX-M-1_ on a 100 kb IncI1 plasmid, respectively.

In this study only one isolate was investigated for the presence of PMQR genes. This isolate lacked mutations in the *gyrA* and *parC* genes in combination with having low MIC values for ciprofloxacin and nalidixic acid of 0.5 mg/L and 8 mg/L, respectively. None of the remaining QREC isolates were investigated for PMQR genes. Consequently, additional PMQR genes cannot be ruled out, especially in the isolates with high ciprofloxacin and nalidixic acid MIC values (≥4 mg/L and >128 mg/L, respectively). All these isolates had two mutations in *gyrA* and one mutation in *parC*. In the NORM-VET monitoring programme there has previously been a sole indication of an isolate encoding PMQR genes based on characteristic MIC spans of ciprofloxacin MIC values between 0.06–1 mg/L and simultaneously nalidixic acid MIC values between 8–32 mg/L. This *E*. *coli* isolate originated from domestically produced chicken meat (S1 Table).

In the present study, most of the isolates were quinolone resistant due to one or two mutations in the *gyrA* gene and one mutation in the *parC* gene. Although isolates belonged to a total of 23 MLVA profiles, the clustering of the isolates into four dominating clusters indicate a relationship between the comprised isolates. This relatedness indicates dissemination of clonally related *E*. *coli* in the turkey production, which is further supported by the serotyping, phylotyping and resistance profiling results.

It is unknown, though, when, why and how QREC in turkey have emerged as there is no selection pressure from the use of quinolones in the Norwegian turkey production. The overall usage of antimicrobial agents in production animals in Norway is low [60]. However, the turkey production in Norway depends on import of breeding animals. It is therefore a possibility that QREC has been imported via breeding animals as is shown for ESC resistant *E*. *coli* in the poultry production [6,61]. This is supported by preliminary results from a Norwegian study of ESC resistant *E*. *coli* found in imported turkey parent flocks (S2 Table) where ESC resistant *E*. *coli* was found in three different flocks. Similar to the findings in this current study, isolates showed co-resistance to ciprofloxacin, nalidixic acid, gentamicin and tetracycline. In addition, the ESC resistance was due to an up-regulation of the chromosomal *ampC* gene caused by mutations in the promoter and attenuator region. Moreover, the isolates had identical MLVA profiles as found for the 13 isolates in the O19/phylogroup B1 cluster in our study. Further, a recent study from Sweden showed that QREC isolates with MLVA profiles similar to that of the above-mentioned cluster were found in both grand-parent birds on arrival to Sweden, as well as in all levels of the broiler production pyramid [62]. Although the Swedish study was performed on broiler and not turkey, it supports our hypothesis that the import of poultry breeding animals may be the source of the quinolone resistant bacteria. This is further followed by vertical transmission downwards in the production pyramid. We cannot rule out other routes of development and dissemination of antimicrobial resistance as it can occur through other unidentified or less characterized mechanisms. For instance, common bacterial stress factors for chromosomal mutations leading to quinolone resistance may be present in the turkey production both nationally and internationally [63].

Our findings of QREC in Norwegian turkey might seem comparable with findings in other European studies. However, in these European studies, quinolone resistance was found in 30–90% of *E*. *coli* tested without applying a selective method [54,64]. In Norway, similar investigations show very low levels of quinolone resistance among the tested *E*. *coli* (1.3% for turkey and 3.6% for broilers) as documented through the NORM-VET monitoring programme [3]. Further, a non-selective procedure testing for quinolone resistance among randomly chosen *E*. *coli* from the same turkey samples as included in the present study found quinolone resistance in 0.9% of the isolates only [31]. QREC is thus commonly found by using selective methods, but most probably they are present in very low numbers among the total *E*. *coli* isolates, similar to the findings of ESC resistant *E*. *coli* in broilers [65].

The presence of QREC in food-producing animals is of concern in many countries. Of special interest are those bacteria harbouring PMQR genes because of their potential to disseminate these genes into human faecal pathogenic bacterial populations. The present study shows that the dominating resistance mechanism among QREC isolates from Norwegian turkey meat is due to chromosomally located mutations, though strains harboring PMQR genes might occur. However, this favourable situation may change over time, and further surveillance is recommended to monitor the situation in the years to come.

## Supporting information

S1 Table Supplemental data file containing the antibiogram and genotype data on the *E*. *coli* containing a plasmid-mediated resistance gene isolated from chicken meat produced in Norway in 2012. (XLSX)

S2 Table Supplemental data file containing metadata and MLVA, PFGE, phenotypic and genotypic results on the seven QREC resistant to beta-lactams from a small study on turkey parent breeding animals imported to Norway in 2011. (XLSX)

S3 Table Supplemental data file containing the results on MLVA, phylotyping, phenotypic serotyping, susceptibility testing and AMR genotyping results on the 78 QREC isolated from Norwegian turkey meat. (XLSX)

S4 Table Supplemental data file containing the metadata on the 78 QREC isolated from Norwegian turkey meat. (XLSX)

S5 Table Supplemental data file containing data on 2013-01-2422 wild type, vector and transconjugant. (XLSX)

S6 Table Supplemental data file containing data on the monovalent and polyvalent antisera used in the phenotypic serotyping. (XLSX)
