# Correction: Occurrence and characterization of quinolone resistant *Escherichia coli* from Norwegian turkey meat and complete sequence of an IncX1 plasmid encoding *qnrS1*

**DOI:** 10.1371/journal.pone.0217321

**Published:** 2019-05-23

**Authors:** The PLOS ONE Staff

There is an error in Table 1. The bold vertical lines denoting epidemiological cut-off values for resistance are missing. The publisher apologizes for the error. Please see the correct Table 1 here.

**Table 1:** Minimum Inhibitory Concentrations (MICs) and antimicrobial resistance in *Escherichia coli* resistant to fluoroquinolones (n = 78) isolated from turkey meat in Norway in 2013.

Substance	Resistance (%)	Distribution of MIC values (mg/L)															
		0.015	0.03	0.06	0.12	0.25	0.5	1	2	4	8	16	32	64	128	256	≥ 512
Ampicillin	57.7							1.3	23.1	16.7	1.3			57.7			
Azithromycin	NA								52.6	39.7	6.4	1.3					
Ciprofloxacin	100					38.5	3.8	1.3		9	41	6.4					
Nalidixic acid	98.7										1.3			3.9	19.2	75.6	
Gentamicin	15.4						39.7	41	3.9				7.7	7.7			
Tetracycline	34.6								65.4					11.5	23.1		
Colistin	0							91	9								
Sulfamethoxazole	60.3										38.5	1.3					60.3
Trimethoprim	34.6					60.3	5.1							34.6			
Chloramphenicol	10.3										89.7				1.3	9	
Cefotaxime	20.5					79.5		12.8	7.7								
Ceftazidime	20.5						79.5		9	11.5							
Meropenem	0		100														
Tigecycline*	0					97.4	2.6										

Bold vertical lines denote epidemiological cut-off values for resistance. NA, cut-off not defined by EUCAST. White fields denote range of dilutions tested for each antimicrobial agent. MIC values higher than the highest concentration tested are given as the lowest MIC value above the range. MIC values equal to or lower than the lowest concentration tested are given as the lowest concentration tested.

*Tentative ECOFF from the EURL-AR (www.eurl-ar.eu).
